# The Lived Experience of Adults With Inflammatory Bowel Disease in Rural Areas: Phenomenological Study

**DOI:** 10.1111/nhs.70058

**Published:** 2025-02-10

**Authors:** Vincenzo Bosco, Caterina Mercuri, Patrizia Doldo, Rita Nocerino, Assunta Guillari, Michele Virgolesi, Teresa Rea, Vincenza Giordano, Silvio Simeone

**Affiliations:** ^1^ Department of Medical and Surgical Sciences, University Hospital Mater Domini Magna Graecia University Catanzaro Italy; ^2^ Department of Clinical and Experimental Medicine University of Catanzaro Magna Graecia Catanzaro Italy; ^3^ Department of Translational Medical Science University of Naples “Federico II” Naples Italy; ^4^ ImmunoNutritionLab at CEINGE—Advanced Biotechnologies University of Naples “Federico II” Naples Italy; ^5^ Department of Public Health University of Naples “Federico II” Naples Italy; ^6^ Department of General Surgery and Women's Health AORN Antonio Cardarelli Naples Italy

**Keywords:** economic burden, inflammatory bowel disease, phenomenology, qualitative study, rural area, social isolation, telemedicine

## Abstract

Inflammatory bowel disease (IBD), including Crohn's disease and ulcerative colitis, is a chronic gastrointestinal condition with a multifactorial etiology. It significantly impacts patients' quality of life, particularly, in rural areas where access to specialized care is limited. Challenges such as increased travel costs, social isolation, and restricted healthcare access are recognized, but their specific impact on rural Italian patients remains underexplored. This study aims to fill this gap. Using Cohen's hermeneutic phenomenological approach, in‐depth interviews were conducted with 17 IBD patients from rural areas. Thematic analysis identified key patterns and themes. Two main themes emerged: “challenges of rural living,” including economic concerns and social isolation, and “communication that bridges distances.” Rural IBD patients face unique challenges extending beyond disease management, with economic hardship and social isolation being prominent. These findings highlight the need for tailored interventions to bridge healthcare gaps and improve the quality of life for rural patients, offering novel insights into their lived experiences in Italy.

**Summary**

- *Economic burden*: People with inflammatory bowel disease (IBD) in rural areas face significant financial challenges due to frequent travel to specialized healthcare centers, adding stress and impacting their overall well‐being and that of their families.
- *Social isolation*: The rural setting exacerbates feelings of social isolation among people affected by IBD due to limited access to healthcare services and misunderstandings or stigma within their communities, which can negatively affect their mental health.
- *Telemedicine as a solution*: Effective communication and the use of telemedicine have shown to bridge the gap in healthcare access for people affected by IBD that live in rural areas, improving their care experience and reducing the emotional and logistical burden associated with managing their condition. By enabling personalized and easily accessible communication, telemedicine further enhances patient engagement, ensuring that care is tailored to individual needs and circumstances.

## 1 Introduction

Inflammatory bowel disease (IBD) is a chronic inflammatory condition of the gastrointestinal tract that primarily includes Crohn's disease and ulcerative colitis. Crohn's disease is characterized by chronic transmural inflammation and ulceration that can affect any part of the gastrointestinal tract, while ulcerative colitis predominantly involves the colon with inflammation limited to the mucosa (Nakase et al. 2021; Rohatinsky et al. 2021).

The etiology of IBD is multifactorial, involving a combination of genetic, immunological, environmental, and microbial factors (Podolsky 2002; Ott et al. 2008; Thomas et al. 2022). These conditions are characterized by unpredictable periods of exacerbation (active disease) and remission (inactive disease) that last a lifetime (Rohatinsky et al. 2021). Recent studies have identified over 240 genetic susceptibility loci for IBD, with new variants discovered in different populations (Cordero et al. 2023). Additionally, alterations in the intestinal microbiota are recognized as key factors in the pathogenesis of IBD, with specific dysbiosis associated with chronic inflammation (Wu et al. 2021).

Common symptoms of IBD include frequent bowel movements, abdominal pain, weight loss, malnutrition, and fatigue (Rubalcava and Gadepalli 2021; Carroll et al. 2019). Extraintestinal manifestations can involve joints, eyes, hepatobiliary tract, and skin, significantly impacting subjects' quality of life (Saez et al. 2023; Greuter and Vavricka 2019; Fourie et al. 2018).

Currently, there is no definitive cure for IBD (Nakase et al. 2021). Treatment goals include inducing and maintaining disease remission and improving the quality of life for affected individuals (Carroll et al. 2019; De Boer et al. 2016), often through the use of immunosuppressive and biological drugs (Privitera et al. 2021). New emerging therapies also focus on modulating the intestinal microbiota through probiotics, fecal microbiota transplantation, and microbial metabolites (Haneishi et al. 2023).

Scientific literature shows considerable variability in the incidence of IBD, with the highest rates in Northern Europe and North America (Loftus 2004). However, in recent decades, newly industrialized countries in Asia, South America, and the Middle East have also reported increasing IBD cases (Kaplan 2015; Simeone et al. 2023).

In 2020, it was estimated that 6.8 million people worldwide suffered from IBD, with a constantly increasing incidence trend (Jairath and Feagan 2020; Shan et al. 2022). The current prevalence of IBD in the Western world is 0.5% of the general population, with a constant increase in new cases each year (Kaplan 2015; Axelrad et al. 2021; Mak et al. 2020). In Italy, although there are no official data, it is estimated that over 200 000 people are affected by IBD. Within 10 years of diagnosis, approximately 50% of those with Crohn's disease and about 20% of those with ulcerative colitis will require surgery (Tsai et al. 2021).

The social and economic impacts associated with the development of IBD are significant (Zhao et al. 2021) with high direct and indirect costs related to healthcare and hospital/ambulatory care (Burisch et al. 2020) surgical interventions (Kaplan 2015), drug therapy (Park et al. 2016), and reduced work productivity (Zhao et al. 2021). Additionally, the cost of reduced quality of life is difficult to measure, invariably affecting subjects and their families (Burisch et al. 2013). Symptoms of IBD influence various aspects of subjects' daily lives, including psychological health, playing a significant role in the disease itself (Fourie et al. 2018). The association of IBD with the development of common mental disorders such as anxiety, depression, and post‐traumatic stress disorder has been well documented (Pothemont et al. 2022; Zheng et al. 2022).

While several quantitative studies have documented the social, psychological, and economic impacts of IBD, qualitative research provides unique insights into how patients perceive and navigate these challenges. For example Fourie et al. (2018) reviewed qualitative studies on living with IBD, identifying themes such as coping with uncertainty, stigma, and disruptions to daily life. Similarly, Byron et al. (2020) conducted a meta‐synthesis of qualitative studies, emphasizing the emotional toll and adaptive strategies employed by patients to manage their conditions. Research by Richard et al. (2020) on rural New Zealand patients highlighted the compounded challenges of geographic isolation and limited access to care, which parallels the experiences of rural Italian IBD patients. However, there remains a paucity of qualitative evidence from non‐Anglophone settings, particularly, in rural areas where unique cultural and systemic factors may shape patient experiences differently.

Despite growing interest in the subjective experiences of individuals with chronic diseases, including IBD (Fourie et al. 2018; Zheng et al. 2022; Byron et al. 2020; Fortin et al. 2004; Didsbury et al. 2016), the international literature highlights several knowledge gaps, particularly, regarding the psychological, social, and economic challenges faced in diverse contexts. While studies have explored the impact of IBD on quality of life, coping strategies, access to care, and mental health (Fourie et al. 2018; Hall et al. 2005; Kemp et al. 2012; Sajadinejad et al. 2012; McCombie et al. 2013), rural contexts remain largely unexplored, particularly, in non‐Anglophone settings like Italy. Our study addresses this gap by exploring the lived experiences of rural Italian IBD patients, using a qualitative hermeneutic phenomenological approach to uncover the deeper meanings of their experiences. This approach allows us to capture the interplay between systemic barriers, such as access to specialized care, and the personal coping mechanisms employed by patients.

The choice of phenomenology, particularly, Cohen's hermeneutic approach, is scientifically justified as it enables an in‐depth exploration of personal experiences while capturing the meanings attributed to them (Flood 2010). This methodology is ideal for nursing research, particularly, when exploring new topics (Cohen et al. 2000). This dual focus aligns with the study's aim of uncovering both the challenges and coping strategies of individuals with IBD, providing a rich and comprehensive understanding of their lived experiences. Cohen's approach has been effectively applied in similar healthcare contexts, making it an appropriate methodological framework for this research.

In many high‐income countries, people with IBD are managed by gastroenterology specialists working in tertiary hospitals located in large urban centers (Richard et al. 2020). In Italy, rural areas are defined as territories with low population density, often distant from urban centers and access to essential services, and constitute approximately 60% of the national territory, housing nearly 17% of the population (Istat 2021). This care model may disadvantage those living in rural areas, where timely access to care can be limited (Richard et al. 2020; Kuenzig et al. 2019).

Existing literature lacks qualitative studies exploring the lived experiences of IBD patients in rural Italy, particularly using a hermeneutic phenomenological approach. This study addresses this gap by providing detailed insights into the unique challenges and coping strategies of this underrepresented population (Fourie et al. 2018; Byron et al. 2020).

This study aims to address this gap by exploring the lived experiences of rural Italian IBD patients, contributing valuable insights to the international literature. Understanding these experiences could inform tailored interventions to safeguard the physical and psychological health of IBD patients and their social networks.

## 2 Materials and Methods

### 2.1 Design

The phenomenology of Cohen et al. (2000) used for this study combines both descriptive phenomenology (Husserlian), which seeks to describe everyday conscious experiences while setting aside preconceived opinions (Craswell 1997), and interpretive phenomenology (Gadamerian), which involves a detailed examination of lived personal experiences. The aim is to generate a deeper understanding of both the lived experiences and the meanings attributed to these experiences (Craswell 1997): “The meanings that participants attribute to their experiences help create the needs they have and how these needs can best be met” (Cohen et al. 2000). The same methodology has been successfully used in previous studies aimed at describing the lived experiences of healthcare professionals and subjects (Simeone et al. 2018; Simeone, Ambrosca et al. 2022; Simeone, Vellone et al. 2022). This study followed the Consolidated Criteria for Reporting Qualitative Research (COREQ) guidelines (Tong et al. 2007).

### 2.2 Sample

A purposive convenience sampling was used for this study. Initial contact with potential participants took place at the University Hospital of Catanzaro, in the Gastroenterology and Interventional Endoscopy Unit of the Mater Domini Hospital, University of Magna Graecia, Catanzaro, Italy. A researcher contacted each potential participant, explaining the nature and purpose of the study. Those who expressed interest in participating in the study were contacted by phone. Informed consent was obtained from all participants. Confidentiality was assured at all stages of the study, and participants were reassured that the final report would not contain any identifying information. Participants were informed that they could withdraw from the study at any time. Their refusal to participate or withdrawal would not affect the quality of care provided in any way. Inclusion criteria were having a confirmed diagnosis of IBD by clinical, endoscopic, histological, and/or radiological assessment; ≥ 18 years old; residing in a rural inland area as defined by the Italian National Institute of Statistics (ISTAT) (Istat 2021), which classifies rural areas based on low population density, remoteness from urban centers, and limited access to essential services; adequate understanding of the Italian language; and signing the informed consent form. Exclusion criteria were the presence of severe preexisting cognitive deficits (e.g., dementia); the presence of terminal or active cancer; organ failure; not living in a rural area; and the desire to withdraw from the study.

### 2.3 Data Collection

In full accordance with the methodology used, “bracketing” was first conducted, which involves setting aside one's ideas and prejudices about the phenomenon being studied. To achieve this, the research team engaged in reflective journaling prior to data collection, documenting their preconceptions and assumptions related to the study's focus. During data collection, these reflections were revisited and updated as necessary, ensuring that personal biases were consciously recognized and mitigated. Bracketing was maintained as an ongoing process during data analysis, with regular team discussions to critically examine potential influences of researchers' perspectives on the interpretation of the data.

A single open‐ended question served as the foundation for interviews; however, follow‐up questions were employed as needed to prompt further elaboration or clarification. These follow‐up questions varied based on participants' responses, allowing for a personalized exploration of their lived experiences.

This reflective technique allows each researcher to “bracket” ideas and preconceptions about the phenomenon. Conducting a reflective technique before data collection and analysis prevents the introduction of biases and interferences related to preconceived ideas that could alter data analysis (Cohen et al. 2000). The interview location and timing were chosen by the participants to facilitate a more transparent description of their experiences. There had been no prior contact between the researcher conducting the interviews and the participants. All interviews were conducted at the participants' homes, without family or friends in the room during data collection, as their presence could influence the interviews. During each interview, the interviewer maintained a friendly and welcoming attitude (Vellone et al. 2012) and did not comment on participants' statements to allow greater freedom of dialogue. To ensure maximum freedom of expression for participants and to keep the “world” of each participant at the center of the research (Polit and Beck 2020), interviews were conducted using a single open‐ended question. Field notes were recorded by the researcher during the interviews, enabling the researcher to record participants' nonverbal communication and personal reflections, as well as reflections on the environment.

The interview concluded when participants seemed to have nothing more to say. Data saturation, or redundancy of analytically developed themes, was reached after 17 interviews (Polit and Beck 2020; Trainor and Graue 2014). Interviews lasted between 20 and 60 min and were audio recorded. To allow researchers to familiarize themselves with the chosen technique, two interviews were conducted but not included in the analysis.

### 2.4 Data Analysis

Interviews were transcribed verbatim and integrated with field notes. Data analysis proceeded iteratively, with each interview being analyzed soon after completion. The preliminary interpretations of the data emerging from the initial analysis should undergo rigorous examination as additional data are gathered. This iterative process fosters the ongoing refinement of these interpretations, which are subsequently reassessed in light of new evidence. Beyond serving as a documented account of the analytic journey, the act of writing itself becomes an integral part of the research process in hermeneutic phenomenology (Simeone, Ambrosca et al. 2022). Transcriptions were first read quickly to gain an overview of participants' experiences. Subsequently, each researcher reread each transcript line by line, assigning indicative themes to the transcriptions. Thematic analysis was conducted concurrently across all completed transcripts to identify patterns and relationships. Saturation was determined when no new themes emerged from consecutive interviews, ensuring a comprehensive understanding of the participants' shared and unique experiences. Researchers compared the analytically developed themes across transcripts to validate consistency and reliability; no discrepancies were found at this stage.

Researchers then utilized member checking by presenting the analytically developed themes to participants for their feedback on the accuracy and resonance of the analysis. Participants confirmed that the themes accurately reflected their experiences, contributing to the credibility of the findings.

The process of collaborative validation was employed, wherein researchers compared the analytically developed themes with participants' feedback to ensure that the analysis accurately represented their lived experiences. This approach enhances the credibility of the findings by incorporating participants' perspectives into the validation process (Tong et al. 2007; Lincoln and Guba 1985). By engaging participants in this reflective process, the study ensures that the identified themes are both accurate and meaningful within the context of their experiences. After drafting the paper in Italian (the native language of the authors), the translation into English was conducted according to the WHO methodology for validating instruments in cultures and languages other than the source language, to ensure that the meaning of the data is maintained. As suggested by the WHO, translators should always aim for the conceptual equivalent of a word or phrase, not a word‐for‐word translation (i.e., not a literal translation) (World Health Organization 2019).

### 2.5 Rigor

Scientific rigor in qualitative research refers to the trustworthiness and validity of the study findings, ensuring that the research process is transparent, credible, and reliable. This includes meeting criteria such as credibility (confidence in the truth of the findings), transferability (the extent to which findings apply to other contexts), dependability (consistency of the findings over time), and confirmability (neutrality of the findings). Adherence to the criteria of Lincoln and Guba (1985) guided our approach to rigor. Credibility was ensured through continuous sampling until data saturation and member checking, where participants validated the themes identified. Dependability was achieved by documenting the research process in detail, while confirmability was supported by maintaining reflexivity and conducting bracketing to minimize bias. Transferability was addressed by providing a rich, detailed description of the participants' context and experiences to allow readers to judge applicability to other settings.

Continuous sampling, with data collection until saturation, ensures credibility. For this study, this credibility corresponds to internal validity and the criterion for ensuring that it examines what it is intended to. The reliability criterion is ensured by using the triangulation technique. This technique involves the participation of two or more researchers in the same analysis to provide multiple observations and conclusions, confirming the results from different perspectives. The confirmability criterion is satisfied by confirming the analytically developed themes with participants and using bracketing by researchers. Finally, the results report a detailed description of participants' experiences and, along with a description of their sociodemographic characteristics, ensure compliance with transferability criteria.

## 3 Results

Seventeen subjects with IBD, eight women and nine men were included in the study. The average age was approximately 50 years. The most represented age group was between 40 and 50 years, with the majority of subjects falling within this range. Most participants were married, with more than one child each. Almost all participants had a high school education level and were employed. More than half of the participants were affected by ulcerative colitis, with a known clinical diagnosis for about 18 years. At the time of the study, about two‐thirds of the enrolled subjects were in a state of disease remission. All participants lived in rural areas, on average more than 70 km from the reference hospital for their care, which was reachable with an average travel time of about 72 min. Main socio‐demographic characteristics of study population are reported in Table 1.

**TABLE 1:** Main socio‐demographic characteristics of study population.

Code	Gender	Age	Marital status	No. of child	Education	Employment	Diagnosis	Diagnostic time	Disease phase	Distance to hospital (in km; in h)
AZ 01	Female	39	Cohabitant	—	High school	Worker	CD	16 years ago	Remission	119; > 2
BY 02	Male	42	Married	—	High school	Casual worker	UC	2 years ago	Flare‐up	111; > 1 h 30 min
CX 03	Male	47	Married	2	Master's degree	Worker	CD	22 years ago	Flare‐up	94; > 1
DW 04	Male	45	Married	—	High school	Worker	UC	12 years ago	Remission	78; > 1
EV 05	Female	52	Married	2	Graduation	Self‐worker	UC	7 years ago	Remission	57; > 1
FU 06	Male	44	Married	1	High school	Self‐worker	CD	4 years ago	Remission	47; > 1
GT 07	Female	62	Married	3	Middle school	Worker	CD	31 years ago	Remission	43; > 0.30 min < 1
HS 08	Female	32	Married	2	High school	Self‐worker	UC	9 years ago	Remission	98; > 1 h 30 min
IR 09	Male	65	Married	2	High school	Self‐worker	UC	3 years ago	Flare‐up	58; > 1
JQ 10	Female	43	Single	—	High school	Self‐worker	UC	21 years ago	Remission	34; > 0.30 < 1
KP 11	Male	64	Married	2	Middle school	Other	UC	40 years ago	Flare‐up	74
LO 12	Female	52	Married	2	Middle school	Other	UC	28 years ago	Remission	77; > 1
MN 13	Female	58	Married	2	High school	Worker	UC	19 years ago	Remission	82; > 1 h 30 min
NM 14	Male	37	Single	1	High school	Worker	CD	29 years ago	Remission	88; > 1 h 30 min
OL 15	Male	67	Married	2	Middle school	Retired	CD	25 years ago	Remission	41; > 0.30 < 1
PK 16	Male	57	Married	2	High school	Worker	CD	30 years ago	Remission	70; > 1 h 30 min
QJ 17	Female	51	Married	2	Graduation	Self‐worker	UC	4 years ago	Flare‐up	59; > 1

Abbreviations: CD, Crohn's disease; UC, ulcerative colitis disease.

The codes generated by the analysis were grouped into categories that were then refined into subthemes. Through iterative discussions among the research team, these were synthesized into main themes that encapsulate the core experiences of the participants.

From the data analysis, two main themes emerged: “Challenges of rural living” and “Communication that bridges distances,” forming the overarching framework for understanding the lived experiences of the participants. The first theme, initially titled “The other side of the coin,” has been rephrased to align with the descriptive/interpretive approach while retaining the original metaphor within the narrative. Participants described the duality of their rural environment—its beauty and tranquility juxtaposed with the hardships imposed by geographic isolation and limited access to specialized care. This duality is reflected in their lived experiences and shaped their perceptions of living with IBD in a rural setting.

The first main theme, “Challenges of rural living,” included two subthemes: (i) “Economic concern,” describing how living in rural areas imposed significant economic challenges, including the costs of travel for medical care and its impact on their families and (ii) “Social isolation,” reflecting feelings of loneliness and stigma due to the lack of understanding in their communities.

The second main theme, “communication that bridges distances,” highlighted participants' appreciation for alternative communication methods, such as telemedicine, which helped reduce their sense of isolation.

By combining subthemes into main themes, the research team ensured coherence and highlighted the interconnectedness of participants' experiences, making the findings both accessible and meaningful while preserving the depth of their narratives.

### 3.1 Challenges of Rural Living

All our participants clearly stated that living in rural areas represented a concern regarding their specific health condition. Specifically, the concern was about timely access to specialized healthcare services and the additional costs required for travel and other expenses. Our participants noted that residing in a rural area meant being far from specialized healthcare services for IBD subjects. This situation generates worry and stress, placing them in a vicious cycle that worsens their clinical condition.

IR 09 said: “Paradoxically, everyone says that living in an area like mine can be fantastic…and let's be clear, it is, everything is on a human scale, so one might ask what's wrong?…well, if you're in good health, it is…it's really beautiful, a paradise on earth, but every day I fear that I might worsen to the point of having to rush to the hospital…and imagine doing kilometers on these roads (sitting upright on the chair, he raises his right arm and then lets it fall resignedly along his body while turning his head towards the door. Meanwhile, his other arm forms an acute angle, resting his hand on his hip)…I always think, will I get there in time or not…and these thoughts, which I always have, accompany me and my illness, making it worse…and that's how I see suffering being here.” EV 05 adds: “I often find myself observing the landscape from the village square and…well, as a child, I used to say how beautiful (sitting in front of us, back straight, feet crossed in front of the chair's legs, speaking calmly, gaze fixed on me, smiling, raising her right arm to indicate the window overlooking the landscape)…now I say…oh my, what isolation, what distance from my hospital (brings both hands to her chest, face becoming serious, voice trembling, and legs retracting back, bringing the crossed feet parallel to the back legs of the chair while leaning forward), what fear…I think every day that the best care for me is far from my world and could become more difficult to reach every day…”…“I have come to what they call the other side of the coin…now I see what I once considered paradise as purgatory…the sun comes out but often I only see darkness…the sun doesn't illuminate as it used to…” She further specifies, “even when I need a check‐up, I have to plan it well in advance and in every detail…I have to figure out how to get to the hospital to be there at the required time, how to be in the best shape for the exam and worry about how to get medications or other things that are not always available here…”.

Finally, OL 15 clarifies: “we now know well that the mind influences the body and I, when I think about the distance between me and the trusted operators…but trusted because they are good, not nice…I mean, because they are specialized in treating my disease, they see many cases…they don't improvise, well, when I think about the possibility of urgent care, an urgent visit well, I think about the distance and wonder if…well (the face becomes serious, eyes moist with tears)…and then my whole situation, my disease worsens…the fact is these thoughts are often present.” Besides the physical distance between where they live and the treatment center for their condition, which worries our participants, there is also the economic aspect. They well describe how this aspect is an integral part of the concern of living in such an area. BY 02 says, “and then, even normally, going there is a cost that affects my life, my family's life…doing exams and, thank God, they (the hospital staff) understand me, they even anticipate my problems (eyes widen, raises both arms to the sky, bringing hands slightly above shoulder height), well it's money and time that I take away from them…and all this makes me angry, makes me feel even worse (adjusts his position on the chair, moving his pelvis back and bringing his back forward. Each hand, closed in a fist, is along the corresponding thigh).” A participant adds (HS 08): “…everything has to be planned in advance, even how to save (turns her head to the left shoulder, lowering it from the previous height. Half‐closes her eyes, forehead slightly lowering. Hands are joined with palms facing up at knee height)…not just the time one has to waste to go there, but needless to say, living here, all the care represents a cost that affects the whole family and this is not fair…having to go to a visit means spending money to go, come back, time taken away from work and so…in short…it's not fair that today things are like fifty years ago, or worse….”

Furthermore, they find that living in a rural area can increase the social isolation in which they find themselves. PK 16 tells us: “at the beginning of my illness, when it was not yet clear what it was, well obviously I didn't go out much, for obvious reasons…and here they started to think badly, making me feel even more alone than I was…they didn't understand me, no one understood me…think that even the doctor…well, even the village doctor didn't understand at first and told me to go to the center where I am now being treated…well, even this made me feel alone and abandoned…” MN 13 clarifies: “the initial symptoms, then the fear of going out, certainly led to isolation…but here it's a small community (the smile on his face tends to diminish, disappear and his serious gaze turns to the balcony) and soon everyone looked at me strangely and avoided me…they were afraid that I might infect them…and so I find myself alone among the lonely (one hand in a fist inside the other, covering it and nervously rubbing the back…suddenly stops and the hands open, falling palm open each on their corresponding thigh)…, with no one to talk to, who can understand because they know what you're going through…”.

### 3.2 Communication That Bridges Distances

All participants found that the alternative communication channels created with the care team certainly represent the strength of this experience, reducing the distances previously described. These methods seem to fully respond to the provision of personalized care appreciated by all. The subjects, who feel truly at the center of the care process, perceive how the healthcare professionals almost anticipate the emergence of needs and meet them effectively and efficiently, countering the perceived isolation and distance.

KP 11 says: “I am not very technological, well, managing via the internet is not familiar to me, yet, despite the many commitments they have, they (the care team) always respond to my messages, calling me back as soon as possible and always trying with me to find the best solution to the various problems that may arise”…“they put me at the center, not them (the face is relaxed, lips hinting at a smile, arms bent, hands on the chest)…and I feel less alone because, it seems strange, but I know they are always there…always.”

AZ 01 adds: “it almost feels like I'm part of the family, with everyone…thanks to this idea of theirs I always feel them close and the nice thing is that there are no distinctions between doctors, nurses, aides, and subjects…we reason together, priorities are explained (sitting, eyes widening and words well‐articulated…one arm retracts, bringing the palm of the hand to the chest, the other extends forward as if indicating a second person), points of view are explained and together the right solution is found.”

BY 02 clarifies: “I don't feel like a subject as I am not there waiting for care imposed from above…or rather, obviously I don't decide the care (stops, opens eyes wider, smiles while hands indicate his chest), but they manage to shape it to my needs often without even needing me to say (eyes widen further, the smile slightly diminishes, hands now on legs with palms up. Shoulders hunch a bit and an expression of amazement appears on his face)…it's like they already know. It is certainly due to the fact that the phone is always free or that an email always receives a response, a message follows with advice or a phone call (hands, with palms up and one inside the other, bump into each other…the rhythmic sound seems to punctuate the words said)…well, let's say that with this method, the distances that scare so much don't really exist.”

## 4 Discussion

This study explored lived experience of subjects with IBD living in rural areas of Italy, who are often underrepresented in the existing literature, highlighting two main themes: “challenges of rural living” with subthemes of “economic concern” and “social isolation”, and “communication that bridges distances.” Participants described the challenges related to the distance from specialized care centers, resulting social isolation, and associated economic concerns, and the coping strategies they employ. The findings from this study provide valuable insights into the unique multifaceted difficulties encountered by this population, which are consistent with and expand upon limited existing literature on the subject.

The first main theme, “Challenges of rural living,” captures the multifaceted difficulties faced by rural IBD patients, with a specific emphasis on the impact of geographic and social isolation. While economic concerns were mentioned as part of participants' experiences, the study primarily focuses on understanding the emotional and relational dimensions of these challenges and the strategies employed to navigate them. This aligns with the descriptive/interpretive phenomenological approach, which seeks to provide a deeper understanding of participants' lived experiences rather than addressing broader systemic issues like economics.

Economic concern, the first subtheme, highlights the significant financial strain imposed by frequent travel to distant specialized healthcare centers. Participants described how these costs impacted their personal and family lives, exacerbating the overall burden of disease management. This finding aligns with previous studies, such as Burisch et al. (2020), which documented substantial direct and indirect costs associated with IBD, including travel expenses and loss of productivity. Similarly, Park et al. (2016) reported that financial strain negatively impacts the quality of life for IBD patients, a sentiment echoed by participants in this study.

Social isolation, the second subtheme, underscores the feelings of loneliness and stigma experienced by participants, stemming from limited social understanding and geographical remoteness. This aligns with the findings of Richard et al. (2020), who observed similar experiences among IBD patients in rural New Zealand, and Peña‐Sánchez et al. (2023), who reported disparities in healthcare access and social support between rural and urban IBD patients. Participants in this study noted that the lack of nearby specialized healthcare further compounded their sense of isolation, a finding that reflects broader systemic challenges in rural healthcare.

In addition, Rohatinsky et al. (2021) reported that rural IBD subjects experienced significant challenges due to limited local services and a lack of healthcare providers knowledgeable about IBD, leading to feelings of isolation and substantial gaps in care. Our findings corroborate these results, with participants expressing frustration over the lack of specialized care in their local areas and the additional burden of traveling long distances to access appropriate healthcare.

Thus, social isolation emerged as a critical issue, significantly impacting the quality of life of rural IBD subjects. The lack of nearby specialized healthcare facilities contributes to feelings of abandonment and loneliness. Participants described the stigma associated with their condition and the misunderstandings from their community, leading to increased isolation. This finding resonates with those of Richard et al. (2020) who studied IBD subjects in rural Southern New Zealand, reporting similar feelings of isolation and the challenges of managing their condition far from specialized care centers. The rural setting, with its smaller and more insular communities, often exacerbates these feelings of isolation, as subjects struggle to find others who understand their experiences and challenges. The disparities between rural and urban IBD subjects in terms of healthcare access have been well‐documented. Peña‐Sánchez et al. (2023) highlighted that rural residents with IBD had fewer gastroenterology visits and a higher risk of hospitalizations compared to urban residents, reflecting significant rural–urban disparities in IBD healthcare access. This disparity results in delayed care and worse health outcomes for rural IBD subjects. Similarly, Benchimol et al. (2018) found that rural IBD subjects in Canada had lower rates of gastroenterologist visits and higher rates of hospitalizations and emergency department visits compared to urban subjects, suggesting significant barriers to specialized care. Rohatinsky et al. (2021) also reported that rural IBD subjects experienced challenges such as limited local services and a lack of healthcare providers with IBD knowledge, leading to feelings of isolation and gaps in care.

Effective communication with healthcare providers emerged as a central coping strategy for participants, helping to mitigate the challenges posed by geographical distance and feelings of isolation. While participants described using alternative communication channels, such as phone calls and emails, the role of telehealth was only tangentially mentioned and not explored in depth. Consequently, this study does not provide sufficient evidence to advocate for the expansion of telehealth services. Instead, the findings emphasize the need for healthcare providers to prioritize consistent and responsive communication strategies that foster a sense of connection and support for rural patients. Telemedicine has been shown to be a valuable tool in managing IBD, providing timely consultations and follow‐ups, thereby reducing the need for physical travel to healthcare facilities (Richard et al. 2020). This method not only helps in continuous monitoring but also in managing acute exacerbations more efficiently (Privitera et al. 2021).

This study provides an in‐depth understanding of the lived experiences of rural IBD patients, using a robust phenomenological methodology. Cohen's approach, which integrates Husserlian descriptive phenomenology with Gadamerian interpretive phenomenology, offers a distinct advantage by capturing both the essence of participants' lived experiences and the meanings they ascribe to those experiences within their social and cultural contexts. The Husserlian component ensures a rigorous description of the phenomenon as experienced by participants, while the Gadamerian component allows for an interpretive layer that situates these experiences within broader existential and relational frameworks (Simeone, Vellone et al. 2022). This dual focus enables a comprehensive and nuanced exploration of complex phenomena, such as the intersection of healthcare access and social isolation in rural IBD patients. Additionally, the use of in‐depth interviews allowed us to explore emotional and psychological aspects that enrich our understanding of the impact of IBD on subjects' lives.

Reflexivity played a critical role in this study, as the researchers acknowledged and examined their own influence on the research process. The use of bracketing was integral to this reflexive practice. By documenting and revisiting preconceptions through reflective journaling and team discussions, the research team reduced the likelihood of these biases shaping the data interpretation. This approach allowed the findings to authentically reflect participants' lived experiences. The team believes that the bracketing process enhanced the credibility of the results by ensuring that emerging themes were grounded in the data rather than influenced by preconceived notions. For example, preconceived assumptions about the prevalence of telemedicine usage in rural areas were set aside, allowing the importance of telemedicine as a coping strategy to emerge organically from participants' narratives. The researchers engaged in reflexive practices throughout the study, including during data collection and analysis, to minimize bias and enhance transparency. For example, the researchers' professional backgrounds in healthcare may have shaped the way participants perceived the interviews, potentially influencing the depth of responses. Conversely, participants' narratives also influenced the researchers, prompting ongoing reflection on their preconceptions and assumptions. This reciprocal dynamic underscores the importance of reflexivity in qualitative research, ensuring that findings authentically reflect the lived experiences of participants while maintaining methodological rigor. However, the study has some limitations. Firstly, the focus on a specific geographical area may limit the generalizability of the results.

## 5 Conclusion

In conclusion, our findings underscore the multifaceted challenges faced by IBD subjects in rural Italy, particularly economic hardships, social isolation, and the psychological toll of the disease. This study highlights the importance of effective communication in addressing the challenges faced by rural IBD patients, particularly, geographic and social isolation. While participants referenced alternative communication methods, this study does not provide sufficient evidence to recommend expanding telehealth services. Instead, the findings underscore the need for consistent, empathetic communication strategies tailored to the unique needs of rural patients. Future research should build upon these findings to further explore how specific interventions can alleviate isolation and enhance the quality of care for this population.

## 6 Relevance for Clinical Practice

Our study reveals key ways to improve care for IBD patients in rural areas. This study highlights the importance of addressing the unique challenges faced by rural IBD patients, particularly, the psychological and social dimensions of geographic and social isolation. The findings emphasize the critical role of tailored interventions, in bridging care gaps and mitigating feelings of isolation. By focusing on these central themes, the study offers actionable insights into improving care delivery and quality of life for this population, while acknowledging that broader systemic issues, such as economic challenges, were not the primary focus of this research. The purpose of this phenomenological approach is to describe and understand the lived experiences of the participants, offering insights into their unique challenges. This study's findings may hold relevance for other rural populations with limited healthcare access, provided there is sufficient similarity in contextual factors, such as distance to healthcare facilities and the associated economic and social burdens. Transferability is supported by the rich descriptions of participants' experiences, allowing readers to assess the applicability of these findings to other contexts. However, the specific cultural and geographical nuances of the Italian rural healthcare system must be taken into account when considering cross‐case transferability. Expanding telehealth services ensures continuous, personalized care without the need for long‐distance travel. Educating the public about IBD through community programs can reduce stigma and build support networks, combating social isolation. Effective communication between patients and healthcare providers, through regular follow‐ups and accessible contact points, enhances patient experience and outcomes. Finally, training local healthcare providers in IBD management ensures patients receive immediate and effective care, reducing the need for extensive travel. These strategies can significantly improve the quality of life and care for rural IBD patients.

## Author Contributions

**Vincenzo Bosco:** methodology, validation, formal analysis, investigation, writing – original draft. **Caterina Mercuri:** conceptualization, methodology, validation, formal analysis, investigation, writing – original draft. **Patrizia Doldo:** conceptualization, data curation, writing – review and editing, visualization, supervision. **Rita Nocerino:** software, writing – original draft, supervision. **Assunta Guillari:** formal analysis, visualization. **Michele Virgolesi:** software. **Teresa Rea:** data curation, writing – review and editing. **Vincenza Giordano:** methodology, validation. **Silvio Simeone:** conceptualization, methodology, writing – original draft, writing – review and editing, visualization, supervision.

## Ethics Statement

The study was approved by Ethics Committee of the Calabria Region, Central Area, with protocol number 119/2022 and was performed in accordance with the Helsinki Declaration (Fortaleza revision, 2004), the Good Clinical Practice Standards (CPMP/ICH/135/95), and with the pertinent European and Italian regulations about privacy. All participants were reassured of the confidentiality of the data. All participants signed an informed consent form, and all were given the opportunity to withdraw from the study at any time during the interview data collection.

## Conflicts of Interest

The authors declare no conflicts of interest.

## Data Availability

The data that support the findings of this study are available from the corresponding author upon reasonable request.

## References

[nhs70058-bib-0001] Axelrad, J. E. , K. H. Cadwell , J.‐F. Colombel , and S. C. Shah . 2021. “The Role of Gastrointestinal Pathogens in Inflammatory Bowel Disease: A Systematic Review.” Therapeutic Advances in Gastroenterology 14: 17562848211004493. 10.1177/17562848211004493. 33868457 PMC8020742

[nhs70058-bib-0002] Benchimol, E. I. , M. E. Kuenzig , C. N. Bernstein , et al. 2018. “Rural and Urban Disparities in the Care of Canadian Patients With Inflammatory Bowel Disease: A Population‐Based Study.” Clinical Epidemiology 10: 1613–1626. 10.2147/CLEP.S178056. 30519110 PMC6233859

[nhs70058-bib-0003] Burisch, J. , T. Jess , M. Martinato , and P. L. Lakatos . 2013. “The Burden of Inflammatory Bowel Disease in Europe.” Journal of Crohn's and Colitis 7: 322–337. 10.1016/j.crohns.2013.01.010. 23395397

[nhs70058-bib-0004] Burisch, J. , H. Vardi , D. Schwartz , et al. 2020. “Health‐Care Costs of Inflammatory Bowel Disease in a Pan‐European, Community‐Based, Inception Cohort During 5 Years of Follow‐Up: A Population‐Based Study.” Lancet Gastroenterology & Hepatology 5: 454–464. 10.1016/S2468-1253(20)30012-1. 32061322

[nhs70058-bib-0005] Byron, C. , N. Cornally , A. Burton , and E. Savage . 2020. “Challenges of Living With and Managing Inflammatory Bowel Disease: A Meta‐Synthesis of Patients' Experiences.” Journal of Clinical Nursing 29: 305–319. 10.1111/jocn.15080. 31631440

[nhs70058-bib-0006] Carroll, M. W. , M. E. Kuenzig , D. R. Mack , et al. 2019. “The Impact of Inflammatory Bowel Disease in Canada 2018: Children and Adolescents With IBD.” Journal of the Canadian Association of Gastroenterology 2: S49–S67. 10.1093/jcag/gwy056. 31294385 PMC6512244

[nhs70058-bib-0007] Cohen, M. Z. , D. L. Kahn , and R. H. Steeves . 2000. Hermeneutic Phenomenological Research: A Practical Guide for Nurse Researchers. Sage Publications.

[nhs70058-bib-0008] Cordero, R. Y. , J. B. Cordero , A. B. Stiemke , et al. 2023. “Trans‐Ancestry, Bayesian Meta‐Analysis Discovers 20 Novel Risk Loci for Inflammatory Bowel Disease in an African American, East Asian and European Cohort.” Human Molecular Genetics 32: 873–882. 10.1093/hmg/ddac269. 36308435 PMC9941836

[nhs70058-bib-0009] Craswell, J. W. 1997. Research Design Qualitative & Quantitative Approaches. Thousand Oaks, CA: SAGE Publications, Inc.

[nhs70058-bib-0010] De Boer, A. G. E. M. , F. Bennebroek Evertsz' , P. C. Stokkers , et al. 2016. “Employment Status, Difficulties at Work and Quality of Life in Inflammatory Bowel Disease Patients.” European Journal of Gastroenterology & Hepatology 28: 1130–1136. 10.1097/MEG.0000000000000685. 27340897

[nhs70058-bib-0011] Didsbury, M. S. , S. Kim , M. M. Medway , et al. 2016. “Socio‐Economic Status and Quality of Life in Children With Chronic Disease: A Systematic Review.” Journal of Paediatrics and Child Health 52: 1062–1069. 10.1111/jpc.13407. 27988995

[nhs70058-bib-0012] Flood, A. 2010. “Understanding Phenomenology: Anne Flood Looks at the Theory and Methods Involved in Phenomenological Research.” Nursing Research 17: 7–15. 10.7748/nr2010.01.17.2.7.c7457. 20222274

[nhs70058-bib-0013] Fortin, M. , L. Lapointe , C. Hudon , A. Vanasse , A. L. Ntetu , and D. Maltais . 2004. “Multimorbidity and Quality of Life in Primary Care: A Systematic Review.” Health and Quality of Life Outcomes 2: 51. 10.1186/1477-7525-2-51. 15380021 PMC526383

[nhs70058-bib-0014] Fourie, S. , D. Jackson , and H. Aveyard . 2018. “Living With Inflammatory Bowel Disease: A Review of Qualitative Research Studies.” International Journal of Nursing Studies 87: 149–156. 10.1016/j.ijnurstu.2018.07.017. 30125834

[nhs70058-bib-0015] Greuter, T. , and S. R. Vavricka . 2019. “Extraintestinal Manifestations in Inflammatory Bowel Disease—Epidemiology, Genetics, and Pathogenesis.” Expert Review of Gastroenterology & Hepatology 13: 307–317. 10.1080/17474124.2019.1574569. 30791773

[nhs70058-bib-0016] Hall, N. J. , G. P. Rubin , A. Dougall , A. P. S. Hungin , and J. Neely . 2005. “The Fight for ‘Health‐Related Normality’: A Qualitative Study of the Experiences of Individuals Living With Established Inflammatory Bowel Disease (IBD).” Journal of Health Psychology 10: 443–455. 10.1177/1359105305051433. 15857873

[nhs70058-bib-0017] Haneishi, Y. , Y. Furuya , M. Hasegawa , A. Picarelli , M. Rossi , and J. Miyamoto . 2023. “Inflammatory Bowel Diseases and Gut Microbiota.” International Journal of Molecular Sciences 24, no. 4: 3817. 10.3390/ijms24043817. 36835245 PMC9958622

[nhs70058-bib-0018] Istat . 2021. Italian National Institute of Statistics. Accessed December 8, 2024. https://www.istat.it/.

[nhs70058-bib-0019] Jairath, V. , and B. G. Feagan . 2020. “Global Burden of Inflammatory Bowel Disease.” Lancet Gastroenterology & Hepatology 5: 2–3. 10.1016/S2468-1253(19)30358-9. 31648974

[nhs70058-bib-0020] Kaplan, G. G. 2015. “The Global Burden of IBD: From 2015 to 2025.” Nature Reviews Gastroenterology & Hepatology 12: 720–727. 10.1038/nrgastro.2015.150. 26323879

[nhs70058-bib-0021] Kemp, K. , J. Griffiths , and K. Lovell . 2012. “Understanding the Health and Social Care Needs of People Living With IBD: A Meta‐Synthesis of the Evidence.” World Journal of Gastroenterology 18: 6240–6249. 10.3748/wjg.v18.i43.6240. 23180944 PMC3501772

[nhs70058-bib-0022] Kuenzig, M. E. , E. I. Benchimol , L. Lee , et al. 2019. “The Impact of Inflammatory Bowel Disease in Canada 2018: Direct Costs and Health Services Utilization.” Journal of the Canadian Association of Gastroenterology 2: S17–S33. 10.1093/jcag/gwy055. 31294382 PMC6512251

[nhs70058-bib-0023] Lincoln, Y. S. , and E. G. Guba . 1985. Naturalistic Inquiry. Newbury Park, CA: SAGE Publications.

[nhs70058-bib-0024] Loftus, E. V. 2004. “Clinical Epidemiology of Inflammatory Bowel Disease: Incidence, Prevalence, and Environmental Influences.” Gastroenterology 126: 1504–1517. 10.1053/j.gastro.2004.01.063. 15168363

[nhs70058-bib-0025] Mak, W. Y. , M. Zhao , S. C. Ng , and J. Burisch . 2020. “The Epidemiology of Inflammatory Bowel Disease: East Meets West.” Journal of Gastroenterology and Hepatology 35: 380–389. 10.1111/jgh.14872. 31596960

[nhs70058-bib-0026] McCombie, A. M. , R. T. Mulder , and R. B. Gearry . 2013. “How IBD Patients Cope With IBD: A Systematic Review.” Journal of Crohn's and Colitis 7: 89–106. 10.1016/j.crohns.2012.05.021. 22718016

[nhs70058-bib-0027] Nakase, H. , M. Uchino , S. Shinzaki , et al. 2021. “Evidence‐Based Clinical Practice Guidelines for Inflammatory Bowel Disease 2020.” Journal of Gastroenterology 56: 489–526. 10.1007/s00535-021-01784-1. 33885977 PMC8137635

[nhs70058-bib-0028] Ott, C. , F. Obermeier , S. Thieler , et al. 2008. “The Incidence of Inflammatory Bowel Disease in a Rural Region of Southern Germany: A Prospective Population‐Based Study.” European Journal of Gastroenterology & Hepatology 20: 917–923. 10.1097/MEG.0b013e3282f97b33. 18794607

[nhs70058-bib-0029] Park, K. T. , R. B. Colletti , D. T. Rubin , B. K. Sharma , A. Thompson , and A. Krueger . 2016. “Health Insurance Paid Costs and Drivers of Costs for Patients With Crohn's Disease in the United States.” American Journal of Gastroenterology 111: 15–23. 10.1038/ajg.2015.207. 26195179

[nhs70058-bib-0030] Peña‐Sánchez, J. N. , J. A. Osei , N. Rohatinsky , et al. 2023. “Inequities in Rural and Urban Health Care Utilization Among Individuals Diagnosed With Inflammatory Bowel Disease: A Retrospective Population‐Based Cohort Study From Saskatchewan, Canada.” Journal of the Canadian Association of Gastroenterology 6: 55–63. 10.1093/jcag/gwac015. 37025513 PMC10071297

[nhs70058-bib-0031] Podolsky, D. K. 2002. “Inflammatory Bowel Disease.” New England Journal of Medicine 347: 417–429. 10.1056/NEJMra020831. 12167685

[nhs70058-bib-0032] Polit, D. , and C. Beck . 2020. Essentials of Nursing Research: Appraising Evidence for Nursing Practice. Lippincott Williams & Wilkins.

[nhs70058-bib-0033] Pothemont, K. , S. Quinton , M. Jayoushe , et al. 2022. “Patient Perspectives on Medical Trauma Related to Inflammatory Bowel Disease.” Journal of Clinical Psychology in Medical Settings 29: 596–607. 10.1007/s10880-021-09805-0. 34292456

[nhs70058-bib-0034] Privitera, G. , D. Pugliese , G. L. Rapaccini , A. Gasbarrini , A. Armuzzi , and L. Guidi . 2021. “Predictors and Early Markers of Response to Biological Therapies in Inflammatory Bowel Diseases.” Journal of Clinical Medicine 10: 853. 10.3390/jcm10040853. 33669579 PMC7922976

[nhs70058-bib-0035] Richard, L. , G. Noller , S. Derrett , et al. 2020. “Patients' Accounts of Living With and Managing Inflammatory Bowel Disease in Rural Southern New Zealand: A Qualitative Study.” BMJ Open 10: e041789. 10.1136/bmjopen-2020-041789. PMC7662529 33184085

[nhs70058-bib-0036] Rohatinsky, N. , I. Boyd , A. Dickson , et al. 2021. “Perspectives of Health Care Use and Access to Care for Individuals Living With Inflammatory Bowel Disease in Rural Canada.” Rural and Remote Health 21, no. 2: 6358. 10.22605/RRH6358. 33820422

[nhs70058-bib-0037] Rubalcava, N. S. , and S. K. Gadepalli . 2021. “Inflammatory Bowel Disease in Children and Adolescents.” Advances in Pediatrics 68: 121–142. 10.1016/j.yapd.2021.05.005. 34243849

[nhs70058-bib-0038] Saez, A. , B. Herrero‐Fernandez , R. Gomez‐Bris , H. Sánchez‐Martinez , and J. M. Gonzalez‐Granado . 2023. “Pathophysiology of Inflammatory Bowel Disease: Innate Immune System.” International Journal of Molecular Sciences 24, no. 2: 1526. 10.3390/ijms24021526. 36675038 PMC9863490

[nhs70058-bib-0039] Sajadinejad, M. S. , K. Asgari , H. Molavi , M. Kalantari , and P. Adibi . 2012. “Psychological Issues in Inflammatory Bowel Disease: An Overview.” Gastroenterology Research and Practice 2012: 1–11. 10.1155/2012/106502. PMC3388477 22778720

[nhs70058-bib-0040] Shan, Y. , M. Lee , and E. B. Chang . 2022. “The Gut Microbiome and Inflammatory Bowel Diseases.” Annual Review of Medicine 73: 455–468. 10.1146/annurev-med-042320-021020. PMC10012812 34555295

[nhs70058-bib-0041] Simeone, S. , R. Ambrosca , E. Vellone , et al. 2022. “Lived Experiences of Frontline Nurses and Physicians Infected by COVID‐19 During Their Activities: A Phenomenological Study.” Nursing and Health Sciences 24: 245–254. 10.1111/nhs.12920. 35049112

[nhs70058-bib-0042] Simeone, S. , C. Mercuri , C. Cosco , V. Bosco , C. Pagliuso , and P. Doldo . 2023. “Enacted Stigma in Inflammatory Bowel Disease: An Italian Phenomenological Study.” Health 11: 474. 10.3390/healthcare11040474. PMC9956300 36833009

[nhs70058-bib-0043] Simeone, S. , G. Pucciarelli , M. Perrone , et al. 2018. “The Lived Experiences of the Parents of Children Admitted to a Paediatric Cardiac Intensive Care Unit.” Heart & Lung 47: 631–637. 10.1016/j.hrtlng.2018.08.002. 30173952

[nhs70058-bib-0044] Simeone, S. , E. Vellone , G. Pucciarelli , and R. Alvaro . 2022. “Emergency Percutaneous Coronary Intervention and Stent Implantation: Patients' Lived Experiences.” Nursing in Critical Care 27: 148–156. 10.1111/nicc.12623. 33780092

[nhs70058-bib-0045] Thomas, J. P. , D. Modos , S. M. Rushbrook , N. Powell , and T. Korcsmaros . 2022. “The Emerging Role of Bile Acids in the Pathogenesis of Inflammatory Bowel Disease.” Frontiers in Immunology 13: 829525. 10.3389/fimmu.2022.829525. 35185922 PMC8850271

[nhs70058-bib-0046] Tong, A. , P. Sainsbury , and J. Craig . 2007. “Consolidated Criteria for Reporting Qualitative Research (COREQ): A 32‐Item Checklist for Interviews and Focus Groups.” International Journal of Quality in Health Care 19: 349–357. 10.1093/intqhc/mzm042. 17872937

[nhs70058-bib-0047] Trainor, A. A. , and E. Graue . 2014. “Evaluating Rigor in Qualitative Methodology and Research Dissemination.” Remedial and Special Education 35: 267–274.

[nhs70058-bib-0048] Tsai, L. , C. Ma , P. S. Dulai , et al. 2021. “Contemporary Risk of Surgery in Patients With Ulcerative Colitis and Crohn's Disease: A Meta‐Analysis of Population‐Based Cohorts.” Clinical Gastroenterology and Hepatology 19: 2031–2045. 10.1016/j.cgh.2020.10.039. 33127595 PMC8934200

[nhs70058-bib-0049] Vellone, E. , G. Piras , G. Venturini , R. Alvaro , and M. Z. Cohen . 2012. “The Experience of Quality of Life for Caregivers of People With Alzheimer's Disease Living in Sardinia, Italy.” Journal of Transcultural Nursing 23: 46–55. 10.1177/1043659611414199. 21807960

[nhs70058-bib-0050] World Health Organization . 2019. “WHO Guidance on Translation: Process of Translation and Adaptation of Instruments,” Accessed May 16, 2024. http://Www.Who.Int/Substance_abuse/Research_tools/Translation/En/.

[nhs70058-bib-0051] Wu, N. , C. Mah , S. Koentgen , et al. 2021. “Inflammatory Bowel Disease and the Gut Microbiota.” Proceedings of the Nutrition Society 80: 424–434. 10.1017/S002966512100197X. 34165053

[nhs70058-bib-0052] Zhao, M. , L. Gönczi , P. L. Lakatos , and J. Burisch . 2021. “The Burden of Inflammatory Bowel Disease in Europe in 2020.” Journal of Crohn's and Colitis 15: 1573–1587. 10.1093/ecco-jcc/jjab029. 33582812

[nhs70058-bib-0053] Zheng, J. , Q. Sun , J. Zhang , and S. C. Ng . 2022. “The Role of Gut Microbiome in Inflammatory Bowel Disease Diagnosis and Prognosis.” United European Gastroenterology Journal 10: 1091–1102. 10.1002/ueg2.12338. 36461896 PMC9752296

